# RasGrf1 and Aging

**DOI:** 10.18632/aging.100331

**Published:** 2011-05-26

**Authors:** Kelvin J. A. Davies, Henry Jay Forman

**Affiliations:** ^1^Ethel Percy Andrus Gerontology Center of the Davis School of Gerontology, the University of Southern California, Los Angeles, CA 90089, USA; ^2^Division of Molecular & Computational Biology, Department of Biological Sciences of the Dornsife College of Letters, Arts & Sciences, the University of Southern California, Los Angeles, CA 90089, USA; ^3^University of California at Merced, Merced, CA 95343, USA

This important article by Borrás et al. demonstrates several interesting and surprising findings [1]. The most intriguing finding was that the complete elimination of normal *RasGrf1* increased both average and maximal longevity independent of a role in cancer. We found this to be surprising because *RasGrf1, a* Ca^2+^/calmodulin-dependent Ras-guanine-nucleotide-releasing factor, would be expected to have a role in normal physiology [2].

The effect of *RasGrf1* deletion on aging, which is also accompanied by lower frailty and retention of motor control, appears to be mediated by greater protection against oxidative damage as observed by lower brain lipid peroxidation, liver protein oxidation and maintenance of the brain and liver glutathione redox potential. We must note that the use of malondialdehyde levels as a measure of overall lipid peroxidation in whole tissues is rather suspect and subject to numerous artifacts; nevertheless, the approximate 25% lower MDA levels in *RasGrf^-/-^* mice (in comparison with wild-type mice) were significant. *RasGrf^-/-^ mice* also exhibited lower levels of oxidized proteins (carbonyl assay) than did wild-type animals which might be due either to lower rates of production, or higher degradation rates of oxidized proteins, or both. It should be noted that old wild-type animals also exhibited lower levels (≈ 20%) of oxidized liver proteins than seen in wild-type mice. This is surprising since levels of oxidized (typically aggregated and cross-linked) proteins usually increase with age, as Proteasome [3-5] and Lon protease [6, 7] activities decline. Indeed, the authors themselves note this expectation in the Results section. Nevertheless, it is impressive that the old *RasGrf1* deletion mutants exhibited almost 30% lower levels of oxidized liver proteins than did the young wild-type mice.

*RASGRF1* is expressed in pancreatic β-cells where it regulates β-cell mass. So the effects on glucose metabolism is unsurprising. *RasGrf1* is also expressed in the hippocampus and hypothalamus is involved in learning and memory. So, one wonders how a human without *RasGrf1* would be able to do those functions and whether living longer and stronger might be accompanied by not remembering why one cared.

Nonetheless, the effect on longevity is remarkable and delving deeper in to how the absence of *RasGrf1* produces this dramatic effect is worthwhile. For example, it would be interesting to determine whether the ability to increase glutathione in response to stress, which declines in aging, is also maintained in the *RasGrf1* knockout mice. Certainly, it would be worth testing the effect of *RasGrf1* deletion on Proteasome and Lon levels and stress-inducibility.

Another interesting point raised by the authors is that *RasGrf1*, which is an exclusively paternal allele imprinted gene, suggests that only the male parent determines the effect of this gene's expression on longevity. In several ways, the *RasGrf1^-/-^* mice metabolically resemble mice fed a calorie-restricted diet. One exciting outcome of these studies is that *RasGrf1* may be a potential target for design of agents that prolong lifespan and healthspan without the obvious difficulty of restricting caloric intake.

## References

[R1] Borras C (2011). Aging.

[R2] Krapivinsky G (2003). Neuron.

[R3] Fucci L (1983). *Proc* Natl Acad Sci U S A.

[R4] Sitte N (2000). FASEB J.

[R5] Friguet B (2000). Ann N Y Acad Sci.

[R6] Bota DA, Davies (2002). Nat Cell Biol.

[R7] Bota DA (2002). FEBS Lett.

